# In Vitro Effects of *Lactobacillus plantarum* LN66 and Antibiotics Used Alone or in Combination on *Helicobacter pylori* Mature Biofilm

**DOI:** 10.3390/microorganisms9020424

**Published:** 2021-02-18

**Authors:** Jianfu Ji, Hong Yang

**Affiliations:** State Key Laboratory of Microbial Metabolism, School of Life Sciences and Biotechnology, Shanghai Jiao Tong University, Shanghai 201100, China; jijianfu@sjtu.edu.cn

**Keywords:** *Lactobacillus plantarum*, *Helicobacter pylori*, biofilm, clarithromycin, levofloxacin

## Abstract

*Helicobacter pylori* is a gastrointestinal pathogen with high prevalence that harms human health. Studies have shown that *H. pylori* can form antibiotic-tolerant biofilms, which may interfere with the efficacy of clinical antibiotic therapy. Probiotics can antagonize planktonic and biofilm pathogen cells and thus may play an auxiliary role in *H. pylori* antibiotic therapy. However, the effects of different probiotic strains and antibiotic combinations on *H. pylori* biofilms need to be further investigated. We determined the cell viability of *H. pylori* mature biofilms after treatment with *Lactobacillus plantarum* LN66 cell-free supernatant (CFS), clarithromycin (CLR), and levofloxacin (LVX) alone or in combination by the XTT method. Biofilm cells were observed by scanning electron microscopy (SEM) and confocal laser scanning microscopy (CLSM). Subsequently, protein and polysaccharide concentrations in biofilm extracellular polymeric substances (EPSs) were quantitatively detected by the Bradford method and the phenol-sulfate method. The results showed that LN66 CFS had an eradication effect on mature *H. pylori* biofilm. When used in combination with CLR, LN66 CFS significantly attenuated the eradication effect of CLR on biofilms; in contrast, when used in combination with LVX, LN66 CFS enhanced the disrupting effect of LVX. We speculate that the different effects of CFS and antibiotic combinations on biofilms may be related to changes in the content of proteins and polysaccharides in EPS and that the combination of CFS and CLR might promote the secretion of EPS, while the combination of CFS and LVX might have the opposite effect. Accordingly, we suggest that supplementation with *L. plantarum* LN66 may provide additional help when therapy involving LVX is used for clinical *H. pylori* biofilm eradication, whereas it may impair CLR efficacy when therapy involving CLR is used.

## 1. Introduction

*Helicobacter pylori* has infected more than half of the world’s population, with infection rates exceeding 80% in China and some Eastern European and South American countries [1]. The close relationship between *H. pylori* infection and the development of chronic gastritis, peptic ulcer disease, and gastric cancer led to its classification as a type I carcinogen by the World Health Organization (WHO) in 1994 [2]. The high prevalence of this pathogen and the harm to human health have prompted most clinical guidelines to recommend *H. pylori* eradication as a good option for the prevention and treatment of a series of gastrointestinal diseases [3]. Although multidrug regimens such as triple therapy have been used in clinical practice, factors such as inappropriate antibiotic use and biofilm formation make eradication an increasingly challenging task.

Biofilm formation may play a crucial role in *H. pylori* long-term colonization and reinfection [4]. Biofilms, as barriers of bacterial cells, can defend against drugs and the host immune system. Secretion of extracellular polymeric substances (EPS), low cellular metabolic activity inside the matrix and upregulation of efflux pump gene expression enhance the tolerance of biofilm cells to harsh environments [5]. In some cases, the antibiotic concentration needed to eradicate bacterial biofilm cells can be 100–1000 times higher than that needed to eradicate planktonic cells [6]. Therefore, the existence of biofilms may increase the drug dose required for *H. pylori* clinical eradication, thus exacerbating the increasing prevalence of drug-resistant strains. Exploration of therapeutic regimens that eradicate *H. pylori* biofilms effectively could enable the development of a new approach for *H. pylori* treatment, providing new strategies for improvement of *H. pylori* eradication rates.

Probiotics are defined as live microorganisms that, when administered in adequate amounts, confer health benefits on the host [3]. Probiotic preparations are deemed safe and can regulate the human gastrointestinal flora and immune system [7]. Recently, studies have indicated that probiotics can inhibit pathogen biofilm formation by inhibiting pathogen growth and interfering with the quorum sensing system or by producing antibacterial substances to disrupt the mature biofilm [8]. In addition, taking probiotic preparations seems to be a good option for eradication of *H. pylori* biofilms. However, clinical results have shown that taking probiotics alone cannot meet the need for *H. pylori* clinical eradication. The use of probiotics adjuncts in combination with certain antibiotic therapies may achieve better therapeutic effects than the use of probiotics alone [3]. This is consistent with the findings of in vitro experiments that probiotic metabolites and certain antibiotics have synergistic antibacterial effects [9]. However, there have been few studies investigating the interactions and mechanisms underlying probiotic–antibiotic combinations from the perspective of *H. pylori* biofilms. We intended to evaluate the eradication effects of *L. plantarum* LN66 cell-free supernatant (CFS), clarithromycin (CLR), and levofloxacin (LVX) on *H. pylori* biofilms, either alone or in combination, and to explore the potential basic mechanisms in order to provide a basis for better *H. pylori* biofilm eradication with reduced antibiotic dosages and improved drug precision in the clinic.

## 2. Materials and Methods

### 2.1. Bacterial Strain, Medium and Growth Conditions

*L. plantarum* LN66 and three reference strain, *L. rhamnosus* GG, *L. paracasei* ATCC334 and *L. reuteri* DSM20016 were provided by Jiaxing Innocul—Probiotics Co., Ltd. (Jiaxing, Zhejiang, China). *H. pylori* ATCC43504, *Escherichia coli* ATCC25922, *Staphylococcus aureus* ATCC29213, *Shigella sonnei* ATCC25931, *Salmonella tyhimurium* ATCC14028 were purchased from the American Type Culture Collection (ATCC). All *Lactobacillus* strains were cultured in modified de Man Rogosa Sharpe broth [10] (tryptone 10 g, beef extract powder 10 g, yeast extract 5 g, glucose 20 g, K_2_HPO_4_ 2 g, ammonium citrate 2 g, MgSO_4_·7H_2_O 0.2 g, MnSO_4_·4H_2_O 0.2 g, brought to a volume of 1 L with distilled water; for solid culture, 1.5% agar powder was added) for 12–24 h at 37 °C. For solid culture, *H. pylori* was inoculated onto Columbia Blood Agar plates containing 10% (*v/v*) defibrinated sheep blood (DSB) for 3–5 d at 37 °C under microaerobic conditions (MGC, Tokyo, Japan). For liquid culture, *H. pylori* was inoculated into Brucella Broth (BB) containing 10% (*v/v*) fetal bovine serum (FBS) for 1–3 d at 37 °C under microaerobic conditions. *E. coli*, *S. aureus*, *S. sonnei*, and *S. tyhimurium* were cultured in Brain Heart Infusion (BHI) medium for 12–24 h at 37 °C.

### 2.2. MICs of Antibiotics

The minimum inhibitory concentrations (MICs) of CLR and LVX (Macklin, Shanghai, China) were determined based on a broth dilution assay with slight modifications [5]. Briefly, *H. pylori* cells and two antibiotics were all diluted in BB containing 10% FBS, and the cells were adjusted to a final concentration of approximately 5 × 10^6^ CFU/mL. Then, 100 μL bacterial suspension was added to the wells of 96-well plates containing two-fold serial antibiotic dilutions to a total volume of 200 μL. The plates were incubated at 37 °C under microaerobic conditions for 3 d, and then the OD_600nm_ values were analyzed using a microplate reader (Tecan, Männedorf, Switzerland). The MIC value was defined as the lowest concentration of the antimicrobial agent that inhibited the growth of bacteria.

### 2.3. Collection of LN66 CFS

To collect the LN66 CFS, the strain was inoculated with modified MRS broth, and the fermentation broth was collected when the cell concentration reached approximately 1 × 10^9^ CFU/mL. Then, the bacterial culture was centrifuged at 4 °C and 7600 rpm for 10 min, and CFS was obtained after filtration with a 0.22 μm filter membrane.

### 2.4. Characterization of L. plantarum LN66

LN66 was identified by 16S rRNA gene sequencing, universal primers 27F and 1492R were used to amplify the 16S rRNA gene. The resistance of LN66 to simulated gastric juice was tested by the method of De Souza [11]. Auto-aggregation and hydrophobicity assay were performed according to Zuo [12]. Antimicrobial activity of LN66 CFS was evaluated by agar-well diffusion assay, five pathogens, *H. pylori* ATCC43504, *E. coli* ATCC25922, *S. aureus* ATCC29213, *S. sonnei* ATCC25931, *S. tyhimurium* ATCC14028 were used as indicator strains. The protein content of LN66 CFS was measured with a Bradford protein assay kit (Sangon, Shanghai, China), the polysaccharide content was measured by the phenol-sulfate method, and the pH value was measured [13,14]. *L. rhamnosus* GG, *L. paracasei* ATCC334, *L. reuteri* DSM20016 were used as positive controls for the above assays.

### 2.5. MIC of LN66 CFS

To determine the MIC value of LN66 CFS, we performed the method described by Pelyuntha with some modifications [15]. The cell concentrations and the culture system were the same as described above for the antibiotic MIC assay except that the two-fold dilutions of antibiotics were replaced with 5–50% (*v/v*) CFS diluted in BB containing 10% FBS. The plates were incubated at 37 °C under microaerobic conditions for 3 d, and then the OD_600nm_ was analyzed using a microplate reader.

### 2.6. FICs of Antibiotics and CFS

The FIC values of antibiotics and LN66 CFS used in combination were determined based on the method described by Yang [16]. Basically, *H. pylori* was inoculated into the culture system for MIC assay, except that the single drug was replaced by different concentrations of CLR + CFS or LVX + CFS. The plates were incubated at 37 °C under microaerobic conditions for 3 d, and then the OD_600nm_ was analyzed using a microplate reader.

FICi = FIC(A) + FIC(B) = (A)/(MICA) + (B)/MIC(B)(1)

FIC values of 0.5 or less, 0.75, 1.0, and 2.0 or more are defined as partially synergistic, additive, indifferent, and antagonistic, respectively.

### 2.7. Construction of Biofilm

An *H. pylori* biofilm was constructed by the colony biofilm method as described by Ge [17]. Briefly, sterile nitrocellulose (NC) membranes (Millipore, Burlington, MA, USA) approximately 1 cm × 1 cm in size were placed on CA containing 10% DSB. The *H. pylori* cell concentration was adjusted to approximately 5 × 10^7^ CFU/mL, and then 20 μL of the bacterial suspension was inoculated onto each NC membrane. After drying the NC membrane for 10 min, the plates were turned over and incubated at 37 °C microaerobic conditions for 3 d to obtain mature *H. pylori* biofilms.

### 2.8. Analysis of CFS Effectiveness after Different Treatments

Analysis of CFS effectiveness after different treatments was based on the method described by Chen with some modifications, and biofilm cell viability was measured by the XTT method [18,19]. Untreated raw CFS was divided into three groups: one was digested with 1 mg/mL proteinase K at 37 °C for 1 h (95 °C for 1 min after incubation to inactivate proteinase K), one was adjusted to pH 6.5 with 1 M NaOH, and one was heat-treated at 115 °C for 20 min. Then, the pretreated CFS and modified MRS broth were diluted to the same concentration as the untreated CFS MIC.

The 2 mL pretreated CFS dilutions, untreated CFS dilutions, and modified MRS dilutions were added to 24-well plates, and then the mature biofilms were transferred to the corresponding wells. The inoculated plates were incubated at 37 °C under microaerobic conditions for 24 h. Then, the suspensions were aspirated, and the NC membranes were washed thrice carefully with PBS. After natural drying, the NC membranes were transferred to new 24-well plates; 600 μL of BB containing 10% FBS and 200 μL of 1 mg/mL XTT solution (containing 5% 0.4 mM menadione) (Macklin) were added, the membranes were incubated at 37 °C under microaerobic dark conditions for 3 h, and the OD_492nm_ values were analyzed with a UV2802 spectrophotometer (UNICO, Shanghai, China).

### 2.9. Drug Sensitivity before and after Biofilm Formation

An *H. pylori* planktonic cell susceptibility assay was carried out. Briefly, the *H. pylori* cell concentration was adjusted to approximately 5 × 10^7^ CFU/mL, and 1 mL of bacterial suspension was added to 24-well plates followed by 1 mL of CLR, LVX, or CFS at different dilutions. In the final system, the *H. pylori* cell concentration was 2.5 × 10^7^ CFU/mL; the CLR or LVX concentration was 1 × MIC, 4 × MIC, 16 × MIC, or 64 × MIC; the CFS concentration was 1 × MIC, 1/2 × MIC, 1/4 × MIC, or 1/8 × MIC. The inoculated plates were incubated at 37 °C under microaerobic conditions for 24 h. Then, the suspensions were centrifuged, and the supernatant was discarded to collect the pelleted cells. The bacteria were washed thrice with PBS and resuspended in 600 μL of BB containing 10% FBS, and cell viability was determined by the XTT method.

An *H. pylori* biofilm cell susceptibility assay was carried out. Briefly, 2 mL of each of the above CLR, LVX, and CFS dilutions was added to 24-well plates, and the mature biofilms were transferred to the corresponding wells. The inoculated plates were incubated at 37 °C under microaerobic conditions for 24 h. The biofilm cell viability was determined as mentioned above.

### 2.10. Effect of CFS Combined with Antibiotics on H. pylori Biofilm Viability

In the separate antibiotic and CFS groups, 2 mL of CLR or LVX diluted to a concentration of 1 × MIC or CFS diluted to a concentration of 1/2 × MIC was added to a 24-well plate. In the antibiotic and CFS combination group, the 2 mL final system contained a CLR concentration of 1 × MIC and a CFS concentration of 1/2 × MIC or an LVX concentration of 1 × MIC and a CFS concentration of 1/2 × MIC. The mature biofilms were transferred to the corresponding wells. The inoculated plates were incubated at 37 °C under microaerobic conditions for 24 h. The biofilm cell viability was determined as mentioned above.

### 2.11. Effect of CFS Combined with Antibiotics on H. pylori Biofilm EPS

Extraction of *H. pylori* biofilm EPS was performed on the basis of the ultrasonic centrifugation method described by Kang with some modifications [20]. The different groups of pretreated biofilms mentioned above were washed thrice with PBS. After natural drying, 1 mL of PBS was added, and the biofilm matrix was sonicated at 37 °C for 15 min and then centrifuged at 4 °C and 10,000 rpm for 30 min. The supernatant was collected for the determination of protein and polysaccharide concentrations in EPS. The protein concentrations were quantitatively measured with a Bradford protein assay kit, and the polysaccharide concentrations were measured by the phenol-sulfate method.

### 2.12. Effect of CFS Combined with Antibiotics on H. pylori Biofilm SEM Observations

The different groups of pretreated biofilms mentioned above were washed thrice with PBS. After natural drying, the biofilms were transferred into 12-well plates and fixed with 2 mL of 2.5% glutaraldehyde solution at 4 °C for 12 h. The fixed biofilms were sequentially dehydrated by adding a gradient of 25%, 50%, 75%, 95%, and 100% ethanol for 15 min per concentration. The dehydrated biofilms were then dried in a CPD 300 automatic critical-point dryer (Leica, Weztlar, Germany). After sputter coating with gold, the biofilms were observed under a S3400II scanning electron microscope (Hitachi, Tokyo, Japan).

### 2.13. Effect of CFS Combined with Antibiotics on H. pylori Biofilm CLSM Observations

The different groups of pretreated biofilms mentioned above were washed thrice with PBS. After drying naturally, the biofilms were transferred into 12-well plates and stained with 2 mL of LIVE/DEAD BacLight Bacterial Viability Kit reagent (Invitrogen, Carlsbad, CA, USA) in a darkroom for 20 min. Then, the dye was aspirated, and the biofilms were washed thrice with PBS before being fixed on coverslips. The biofilms were observed under a TCS SP8 STED 3X laser confocal microscope (Leica, Weztlar, Germany).

### 2.14. Effect of CFS Combined with Antibiotics on H. pylori Biofilm Virulence Gene Expression

Total RNA was extracted using a TransZol Up Plus RNA Kit (Transgen, Beijing, China). Then, cDNA was synthesized using a PrimeScript RT Reagent Kit with gDNA Eraser (Takara, Kyoto, Japan). Finally, TB Green Premix Ex Taq™ II (Takara, Kyoto, Japan) was added for fluorescence detection on a qTOWER3G quantitative PCR apparatus (Analytik Jena, Jena, Germany). The primers used for qPCR are listed in Table 1. The expression of each gene was normalized to the 16S rRNA expression as an internal control, and the relative expression was calculated using the 2^−∆∆CT^ method.

### 2.15. Effect of CFS Combined with Antibiotics on H. pylori Biofilm Live Cell Dispersion

The effects of different combinations on biofilm live cell dispersion were assessed by measuring cell viability in suspension. In brief, 600 μL suspensions were taken from wells containing the pretreated biofilms in the different groups mentioned above, and the cell viability was determined by the XTT method.

### 2.16. Effect of CFS Combined with Antibiotics on H. pylori Planktonic Cell Viability

The *H. pylori* cell concentration was adjusted to approximately 5 × 10^7^ CFU/mL, and 1 mL of bacterial suspension was added to 24-well plates followed by 1 mL of CLR, LVX, or CFS dilution separately or in combination to achieve the abovementioned combinations and concentrations for the biofilm assay. The inoculated plates were incubated at 37 °C under microaerobic conditions for 24 h. The planktonic cell viability was determined as mentioned above.

### 2.17. Statistical Analysis

Each assay was performed in triplicate, and the data are expressed as the means ± standard deviations. The differences between the groups were examined by one-way analysis of variance (ANOVA) followed by Turkey’s Multiple Comparison test using Minitab 18.1 software (Minitab, State College, PA, USA). A *p*-value of <0.05 was considered to indicate statistical significance.

## 3. Results

### 3.1. MICs and Characterization of LN66

The MIC values of CLR, LVX, and LN66 CFS were determined by the broth dilution method. As shown in Table 2, the MIC values of CLR and LVX were 0.063 and 0.250 μg/mL, respectively.

LN66 was identified as *L. plantarum* by 16S rRNA gene sequencing (Supplementary Figure S1). The strain can survive at pH 2.0 simulated gastric juice for 2 h, and holds a moderate hydrophobicity and auto-aggregation capabilities compared to the reference strains (Supplementary Figures S2 and S3).

After cultivated to approximately 1 × 10^9^ CFU/mL, LN66 CFS was collected. LN66 CFS had significant inhibitory activities against *H. pylori*, *S. aureus* and *S. sonnei*, compared to MRS medium and the reference strain, and shows the capability of antagonizing *E. coli* and *S. tyhimurium* (Supplementary Table S1).

Results showed that CFS diluted with BB containing 10% FBS to a final concentration of 12.5% was the lowest dilution that could inhibit the growth of *H. pylori*, so the MIC value of LN66 CFS was defined as 12.5%.

Moreover, the FICs, see in Table 3, shows that the effect between CLR + CFS is antagonist, and the effect between LVX + CFS is additive.

### 3.2. The Eradication Effect of LN66 CFS on H. pylori Mature Biofilms Is Derived Mainly from Heat-Stable Peptides

The viability of *H. pylori* biofilm cells after 24 h of CFS treatment was determined by the XTT method, with a higher OD_492nm_ value representing greater biofilm viability. We first divided the LN66 CFS into three groups that were heat-treated, neutralized and proteinase K-treated. Then, untreated CFS, pretreated CFS, and modified MRS broth were all diluted to 12.5%, and the *H. pylori* mature biofilms in each group were incubated for 24 h.

**Figure 1:**
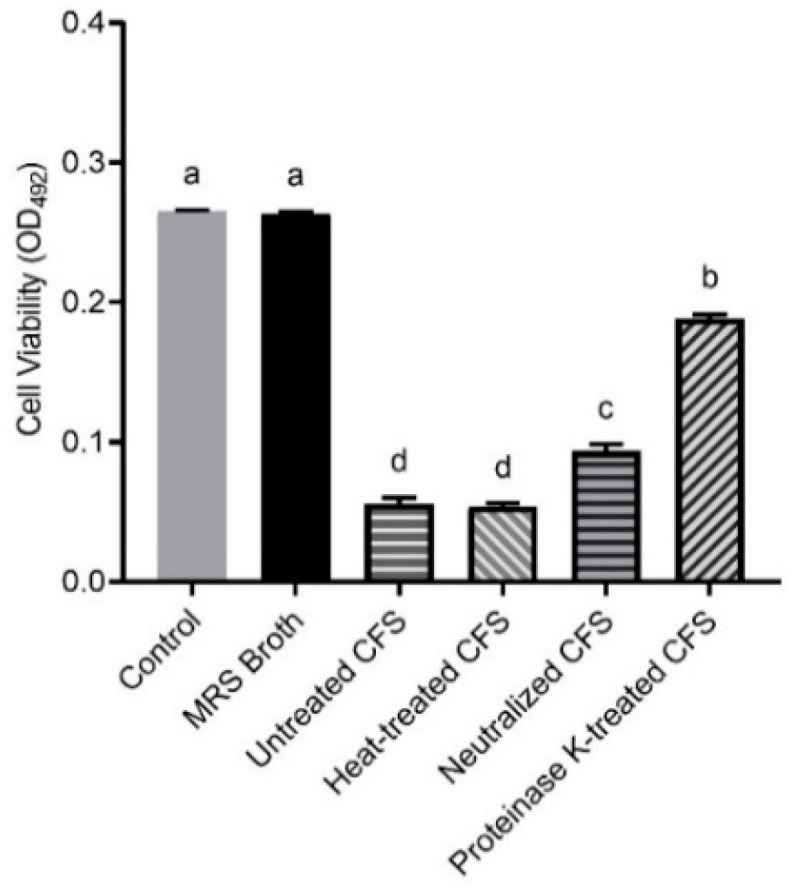
Eradication effects of untreated LN66 CFS, three types of pretreated CFS and modified MRS broth at 12.5% dilutions on *Helicobacter pylori* mature biofilms. The experimental data are expressed as the mean ± standard deviation (*n* = 3), and groups marked with different superscript letters are significantly different (*p* < 0.05).

The results showed that *L. plantarum* LN66 CFS had an eradication effect on *H. pylori* biofilms, and this effect was not derived from the modified MRS broth but from the LN66 metabolized derivatives. Additionally, although the LN66 CFS hold the lowest protein, polysaccharide contents and pH value compared to MRS medium and the references’ CFS (Supplementary Table S2), the ability of LN66 CFS to eradicate *H. pylori* biofilm did not mainly depend on its low pH value. As shown in Figure 1, the OD492 nm measured at 24 h in the control group was 0.265 ± 0.002, which was not significantly different (*p* < 0.05) from the OD492 nm in the modified MRS group, whereas the viability was significantly lower in the four CFS groups than in the control group. In addition, different pretreatment methods caused different impacts on the eradication capacity of CFS. The only pretreatment that did not weaken the CFS eradication capacity was the heat treatment; the heat-treated group had the strongest biofilm eradication capacity among pretreatment groups, exhibiting the same capacity as the untreated group; the heat-treated and untreated groups had OD492 nm values of 0.054 ± 0.002 and 0.056 ± 0.004, respectively. The remaining two pretreatment groups had significantly weaker eradication capacity than the untreated group. Proteinase K treatment reduced the CFS eradication capacity to a great extent, resulting in an OD492 nm of 0.188 ± 0.003. Neutralization, on the other hand, caused CFS to lose less of its eradication capacity, resulting in an OD492 nm of 0.093 ± 0.005.

**Figure 2:**
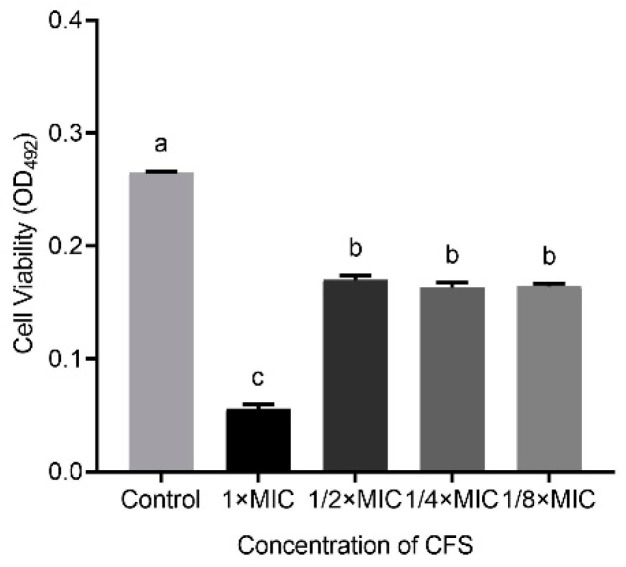
Eradication effects of untreated LN66 CFS at 1 × MIC, 1/2 × MIC, 1/4 × MIC, and 1/8 × MIC dilutions on *H. pylori* mature biofilms. The experimental data are expressed as the mean ± standard deviation (*n* =3), and groups marked with different superscript letters are significantly different (*p* < 0.05).

In addition, Figure 2 shows that the capacity of LN66 CFS to eradicate *H. pylori* biofilm was not significantly weakened (*p* < 0.05) when the CFS was diluted from 1/2 × MIC to 1/8 × MIC. When the concentration decreased from 1 × MIC to 1/2 × MIC, the eradication capacity of CFS decreased significantly, but when the concentration continued to drop to 1/4 × MIC or 1/8 × MIC, the eradication capacity of CFS remained unchanged.

### 3.3. H. pylori Sensitivity to High Concentrations of CLR and LVX Is Reduced after Mature Biofilm Formation

To investigate the sensitivity of *H. pylori* to CLR and LVX before and after biofilm formation, we tested four groups with different dilutions (1 × MIC, 4 × MIC, 16 × MIC, and 64 × MIC) of each antibiotic. Cell viability after 24 h of treatment with different concentrations of CLR or LVX was measured by the XTT method described above.

**Figure 3:**
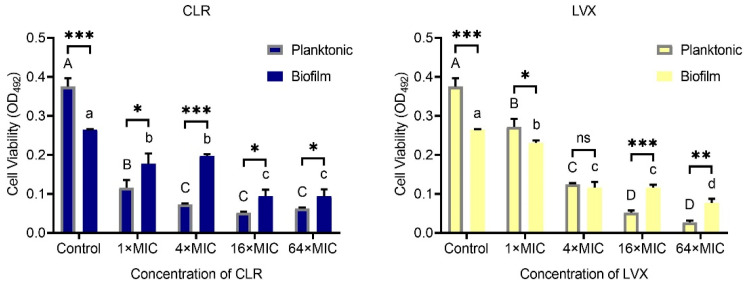
Eradication effects of CLR and LVX at 1 × MIC, 4 × MIC, 16 × MIC, and 64 × MIC dilutions on *H. pylori* planktonic cells and mature biofilms. The experimental data are expressed as the mean ± standard deviation (*n* = 3), and differences in viability between planktonic cells and mature biofilm cells with the same concentration of the same antibiotic are expressed with asterisks (*); * (*p* < 0.05), ** (*p* < 0.01), and *** (*p* < 0.001), ns. non-significant. Differences in viability among planktonic cells with different concentrations of the same antibiotic are expressed with different capitalized superscript letters (*p* < 0.05). Differences in viability among biofilm cells with different concentrations of the same antibiotic are expressed with different lowercase superscript letters (*p* < 0.05).

As shown in Figure 3, *H. pylori* was less sensitive to high concentrations of CLR and LVX after the formation of biofilms than before the formation of biofilms. At a 1 × MIC or 4 × MIC dilution, the capacity of CLR to eradicate *H. pylori* biofilm cells was significantly weaker than the capacity of CLR to eradicate the corresponding planktonic cells (*p* < 0.05). In the LVX group, biofilm cell viability was significantly lower than planktonic cell viability at a 1 × MIC dilution, and there was no significant difference in viability between the two types of cells at a 4 × MIC dilution. Furthermore, we found that the *H. pylori* biofilm viability after 24 h of 16 × MIC or 64 × MIC CLR or LVX treatment was significantly higher than that of the corresponding planktonic cells. Surprisingly, the biofilm cell viability of the control group was significantly lower than the planktonic cell viability of the control group, which may have been due to the specificity of the colony biofilm construction approach used in our study. Unlike microtiter plate biofilm cultured in broth medium, colony biofilm is established in agar medium, thus the colony biofilm, with large biomass, needs to adapt to the liquid environment, whereas the planktonic assay inoculated *H. pylori* culture suspension into the BB broth directly [21].

### 3.4. Combination of CFS and Different Antibiotics Can Influence the Viability of H. pylori Mature Biofilms

Based on the fact that *H. pylori* improves its tolerance to CLR and LVX after biofilm formation, and that probiotic metabolites may improve the efficacy of antibiotic, we investigate the eradication effects of CFS and CLR, LVX alone, or CFS and antibiotics in combination on mature biofilms [16]. We used 1 × MIC dilutions of antibiotics, the CFS dilution adjusted to 1/2 × MIC, which was 6.25%.

**Figure 4:**
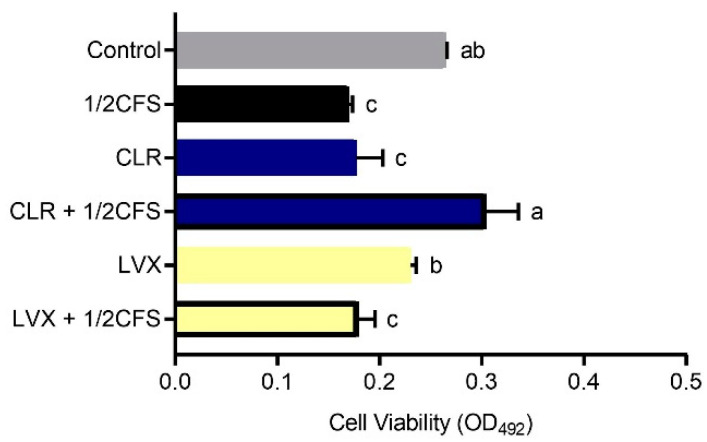
Eradication effects of a 1/2 × MIC dilution of CFS, 1 × MIC dilutions of CLR and LVX alone, and the same dilutions of CFS and antibiotic combinations on *H. pylori* mature biofilms. The experimental data are expressed as the mean ± standard deviation (*n* = 3), and groups marked with different superscript letters are significantly different (*p* < 0.05).

The biofilm viability of all experimental groups was significantly lower (*p* < 0.05) than that of the control group, except for the CLR + 1/2CFS group, for which the OD_492nm_ was significantly higher than the 0.265 ± 0.002 of the control group, see Figure 4. The strongest eradication capacity appeared in the 1/2CFS group, and the weakest eradication capacity was observed for the CLR + 1/2CFS group; the OD_492nm_ values of these groups were 0.170 ± 0.004 and 0.304 ± 0.032, respectively. In the antibiotic-only groups, CLR was significantly more powerful than LVX in eradicating biofilms. However, when antibiotics were used in combination with CFS, the results were strikingly different. CLR, which had stronger eradication capacity than LVX when used alone, completely lost its ability to eradicate biofilms in combination with CFS, and the OD_492nm_ was significantly higher in the combination group than in the CLR group and the 1/2CFS group. The eradication ability of the LVX + 1/2CFS group was significantly better than that of LVX alone, with OD_492nm_ values of 0.179 ± 0.016 and 0.230 ± 0.006, respectively. However, the eradication ability of the LVX + 1/2CFS group was not significantly different from that of the 1/2CFS group.

### 3.5. Combination of CFS and Different Antibiotics Can Affect the Protein and Polysaccharide Content of H. pylori Mature Biofilm EPS

The concentration and combinations of CFS and antibiotics were referred to the viability assay. To determine the content of proteins and polysaccharides in biofilm EPS, we used the Bradford method and the phenol-sulfate method, respectively, and the content was calculated according to the standard curves.

**Figure 5:**
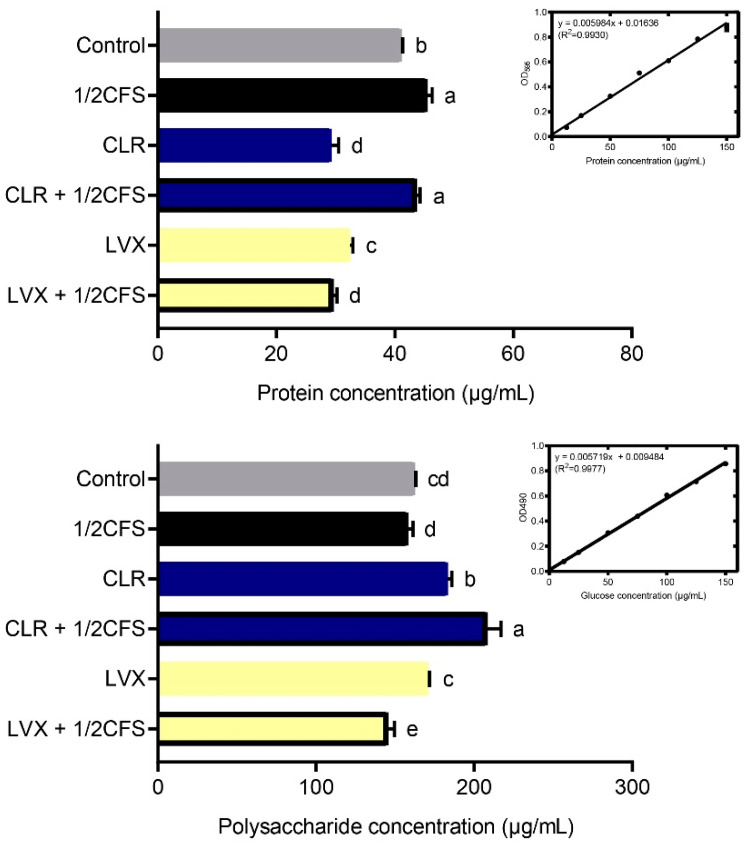
Effects of a 1/2 × MIC dilution of CFS, 1 × MIC dilutions of CLR and LVX alone, or the same dilutions of CFS and antibiotic combinations on the protein and polysaccharide content of *H. pylori* mature biofilm EPS. The experimental data are expressed as the mean ± standard deviation (*n* = 3), and groups marked with different superscript letters are significantly different (*p* < 0.05).

As shown in Figure 5, the protein content measured in most experimental groups was significantly lower (*p* < 0.05) than that in the control group; however, the concentrations measured in the 1/2CFS and CLR + 1/2CFS groups were significantly higher than that in the control group. The 1/2CFS group had the highest protein content of 45.446 ± 0.841 μg/mL, and the control group had a content of 41.101 ± 0.193 μg/mL. The lowest content, 29.291 ± 1.205 μg/mL, was in the CLR group. When antibiotics were used alone, CLR had a stronger effect than LVX with regard to reducing the protein content of EPS. However, during combination treatment, we found that CFS significantly attenuated the effect of CLR on the reduction in protein content in EPS, and the protein concentration in this group was even higher than that in the control group. The opposite results were observed for the LVX + 1/2CFS group, with significantly lower protein content in the LVX + 1/2CFS group than in the LVX group or the 1/2CFS group, suggesting a possible additive effect of CFS and LVX.

The polysaccharide results were quite different from the protein results. The polysaccharide content measured in the LVX + 1/2CFS group was significantly lower than that in the control group (*p* < 0.05), but the polysaccharide content measured in the 1/2CFS group was not significantly different from that in the control group, and the remaining groups possessed significantly higher polysaccharide levels than the control group. The CLR + 1/2CFS group had the highest polysaccharide content among all groups at 208.38 ± 8.51 μg/mL, while the LVX + 1/2CFS group possessed the lowest polysaccharide concentration of 145.43 ± 4.17 μg/mL. Among the antibiotic-only groups, CLR had a weaker effect than LVX with regard to reducing the polysaccharide content in biofilm EPS. In the combination treatment, the CLR + 1/2CFS group exhibited the greatest elevation in polysaccharide content in EPS, with a higher content than the 1/2CFS group and the CLR group. CFS intensified the LVX-mediated reduction in polysaccharide content; the polysaccharide concentration in the LVX + 1/2CFS group was significantly lower than that in the LVX group and the 1/2CFS groups.

### 3.6. SEM Observation of H. pylori Mature Biofilm Cell Morphology

**Figure 6:**
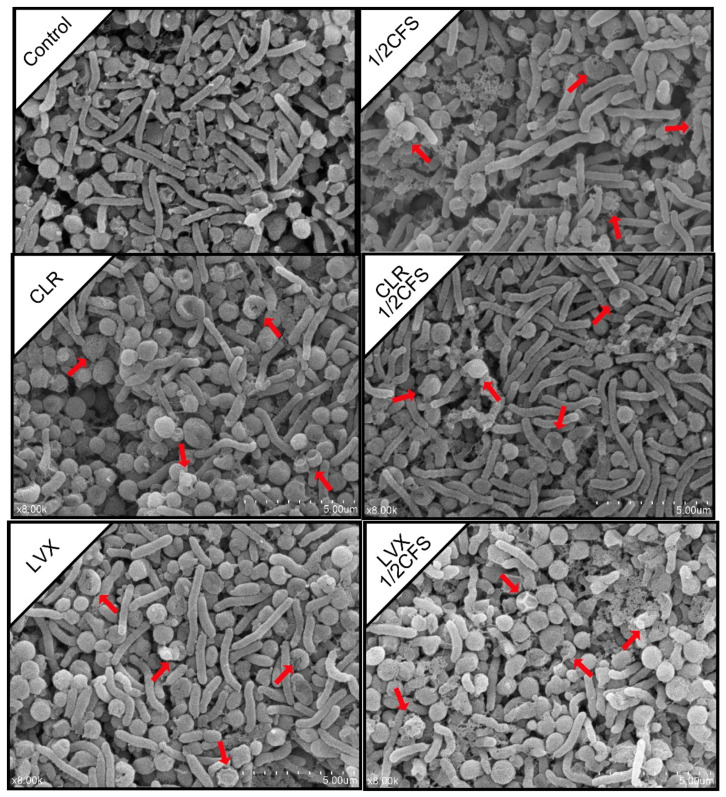
SEM images of a 1/2 × MIC dilution of CFS, 1 × MIC dilutions of CLR and LVX alone, and the same dilutions of CFS and antibiotic combinations on *H. pylori* mature biofilm cell morphology. The magnification is 8000×, and the scale bar is 5.00 μm. Red arrows indicate cells with morphological damages.

We used SEM to observe the morphology of *H. pylori* biofilm cells after 24 h of treatment. As shown in Figure 6, coccoid *H. pylori* cells were observed in all groups, but the CLR + 1/2CFS group presented the most spiral cells. In addition, *H. pylori* cells in different groups showed varying degrees of morphological damages.

Spiral and coccoid cells were more evenly distributed in the control group. The proportion of spiral cells in the 1/2CFS group was higher than that of the coccoid cells, and most coccoid cells in this group were damaged, while only a few spiral cells were damaged. In the CLR group, the proportion of spiral cells was slightly lower than that of the coccoid cells, and all the cells remained essentially intact, with the exception of a few coccoid cells that were damaged. There were many more spiral cells in the CLR + 1/2CFS group than in the CLR group, the 1/2CFS group, and the control group. The spiral cells in the CLR + 1/2CFS group all maintained cell integrity, while few of the remaining coccoid cells were damaged. Spiral and coccoid cells were evenly distributed in the LVX group, and only a small proportion of coccoid cells were damaged. More coccoid cells were present in the LVX + 1/2CFS group than in the LVX group and the 1/2 × CFS group. The cells in this group showed severe damage, with some coccoid cells completely broken.

### 3.7. CLSM Observation of H. pylori Mature Biofilm Structure

**Figure 7:**
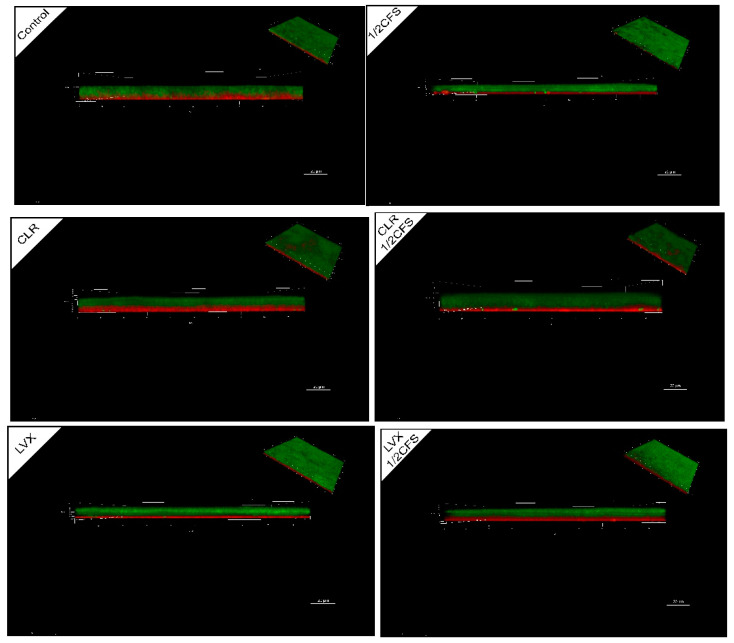
CLSM images of a 1/2 × MIC dilution of CFS, 1 × MIC dilutions of CLR and LVX alone, or the same dilutions of CFS and antibiotic combinations on *H. pylori* mature biofilm structure. All cells stained with SYTO 9 show green fluorescence, and dead cells stained with propidium iodide (PI) show red fluorescence. The bottom layer of the biofilm in the main view is close to the NC membrane surface. The front view shows the horizontal observations along the x-y plane, and the scale bar is 20.00 μm.

The overall structure of *H. pylori* biofilm after 24 h of treatment was observed by CLSM. In Figure 7, green fluorescence represents all cells, and red fluorescence represents dead cells. A layer of dead cells appeared at the bottom of the biofilm near the NC membrane in all groups, and the thickness of the dead cell layer varied greatly among different groups. As shown in Table 4, the thickest live cell layer of experimental group was observed in the CLR + 1/2CFS group while the thinnest was observed in the 1/2CFS group, CLR group and the LVX + 1/2CFS group. The thickest dead cell layer was observed in the CLR group, and the thinnest was observed in the control group, 1/2CFS group and the LVX group. The cell staining images and the XTT results were almost identical in all groups, indicating that groups with thicker live cell layers possess greater viability and that those with thinner live cell layers possess lower viability. The live cell layer in the CLR group was evidently thinner than that in the CLR + 1/2CFS group, and the LVX group had a significantly greater live cell layer thickness than the LVX + 1/2CFS group.

### 3.8. Combined Treatment with CFS and Different Antibiotics Can Affect Virulence Gene Expression in H. pylori Biofilm Cells

**Figure 8:**
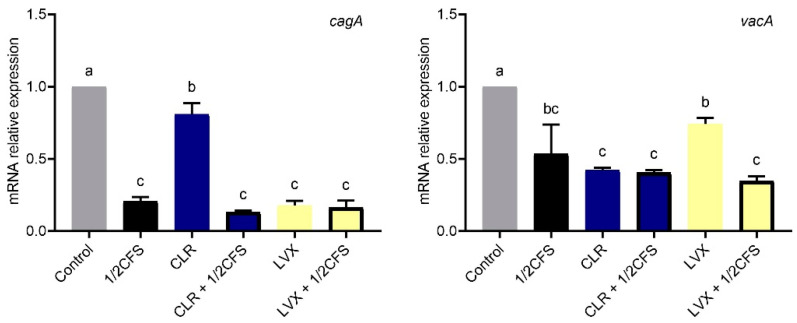
Effects of a 1/2 × MIC dilution of CFS, 1 × MIC dilutions of CLR and LVX alone, or the same dilutions of CFS and antibiotic combinations on *cagA* and *vacA* relative expression in *H. pylori* biofilm cells. The experimental data are expressed as the mean ± standard deviation (*n* = 3), and groups marked with different superscript letters are significantly different (*p* < 0.05).

For *H. pylori* biofilm cells after 24 h of treatment, we determined the relative expression of the *cagA* and *vacA* genes. As shown in Figure 8, the relative expression of the *cagA* gene was significantly downregulated (*p* < 0.05) in all experimental groups compared with the control group. Among the experimental groups, the CLR group showed the weakest downregulation, whereas the rest of these groups were not significantly different from each other. The relative expression of *cagA* was significantly reduced by combined treatment with CLR and CFS compared with CLR alone; the relative expression was 0.807 times in the CLR group and 0.132 times in the CLR + 1/2CFS group. In addition, we found significant downregulation of *vacA* expression in all experimental groups compared to the control group. Among the combined CFS and antibiotic groups, the CLR + 1/2CFS group was not significantly different compared with the CLR group. The relative expression of the *vacA* gene in the LVX + 1/2CFS group was significantly lower than that in the LVX group.

### 3.9. Combined Treatment with CFS and Different Antibiotics Can Inhibit Live H. pylori Mature Biofilm Cell Dispersion

**Figure 9:**
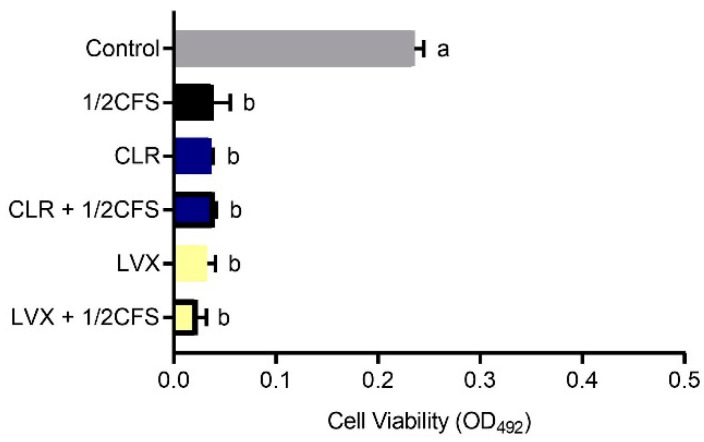
Effects of a 1/2 × MIC dilution of CFS, 1 × MIC dilutions of CLR and LVX alone, or the same dilutions of CFS and antibiotic combinations on live *H. pylori* mature biofilm cell dispersion. The experimental data are expressed as the mean ± standard deviation (*n* = 3), and groups marked with different superscript letters are significantly different (*p* < 0.05).

In addition to determining the eradication effects of different combinations on *H. pylori* biofilms, we also evaluated the inhibitory effects of these combinations on live cell dispersion from biofilms by measuring the viability of cells in suspension after 24 h of biofilm eradication. As shown in Figure 9, the cell viability in the control group was highest, and the OD_492nm_ values measured in the remaining groups were all significantly lower (*p* < 0.05) than the value in the control group.

### 3.10. Combined Treatment with CFS and Different Antibiotics Can Influence the Viability of H. pylori Planktonic Cells

**Figure 10:**
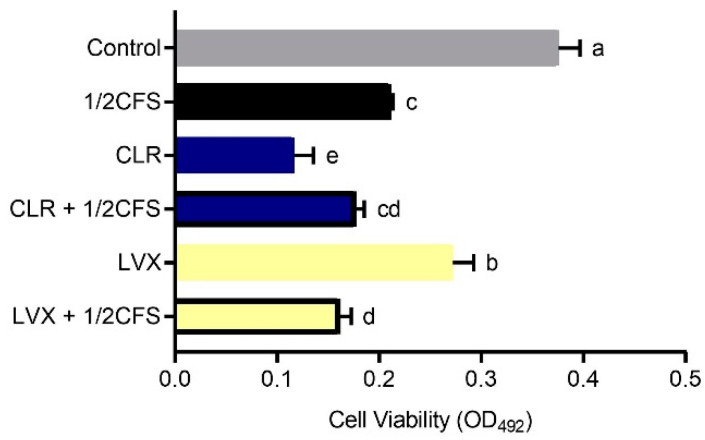
Effects of a 1/2 × MIC dilution of CFS, 1 × MIC dilutions of CLR and LVX alone, or the same dilutions of CFS and antibiotic combinations on *H. pylori* planktonic cell eradication. The experimental data are expressed as the mean ± standard deviation (*n* = 3), and groups marked with different superscript letters are significantly different (*p* < 0.05).

Finally, we determined the eradication effects of different combinations on *H. pylori* planktonic cells, and the results were similar to those measured in the biofilm cell assay. As shown in Figure 10, the cell viability measured in all experimental groups was significantly lower than that in the control group (*p* < 0.05). Among the antibiotics used alone, CLR was significantly more effective than LVX in eradicating planktonic cells. However, the cell viability of the CLR + 1/2CFS group was not significantly different from that of the LVX + 1/2CFS group. In addition, the viability of the CLR + 1/2CFS group was significantly higher than that of the CLR group, while the viability of the LVX + 1/2CFS group was significantly lower than that of the LVX-only group.

## 4. Discussion

It is well known that *H. pylori* infection is associated with the development of a variety of gastrointestinal diseases. Clinical *H. pylori* eradication regimens usually use multidrug combination therapy, but the existence of multidrug-resistant strains and biofilms sometimes makes it difficult for therapies to achieve the optimal effects [22]. The emergence of multidrug-resistant strains may be related to inappropriate antibiotic use, and stubborn biofilms are natural barriers constructed by bacteria themselves against drugs and the host immune system [4]. A biofilm is defined as a bacterial population adhered to a substrate that is encapsulated by a layer of EPS, which is composed mainly of polysaccharides, proteins, and extracellular DNA (eDNA) secreted by bacteria [5]. Since the cells inside the biofilm are surrounded by EPS to form one entity, the total size exceeds the size that can be engulfed by immune cells such as neutrophils, thus enabling defense against the host immune system [23]. EPS also forms a barrier blocking antimicrobial drugs from reaching the cells inside. Cells encapsulated within EPS tend to have low metabolic rates accompanied by drug-efflux gene upregulation; these characteristics improve the resistance of the cells to death caused by antimicrobial drugs [5]. In vitro studies have demonstrated that the presence of a biofilm significantly increases the antibiotic resistance of *H. pylori*, with some strains exhibiting 4-fold and 40-fold higher MICs for clarithromycin and amoxicillin, respectively, among biofilm cells than among planktonic cells [24]. In our study, we investigated the sensitivity of *H. pylori* ATCC43504 before and after biofilm formation to CLR and LVX, two antibiotics that have been reported to be increasingly resisted in recent years [25]. As shown in Figure 3, the sensitivity of the *H. pylori* strain to high concentrations of CLR and LVX decreased significantly after mature biofilm formation. Unfortunately, biofilm cells may be prevalent in the gastric mucosae of *H. pylori*-infected patients. Coticchia et al. detected *H. pylori* biofilm density in the gastric mucosa of patients by endoscopy and SEM, and the results showed that the average biofilm coverage in urease test-positive patients was approximately 97.3%, while the coverage in urease test-negative patients was merely 1.64% [26]. Thus, prevalent and stubborn biofilms may be pivotal interfering factors in *H. pylori* clinical eradication.

Probiotics, which are emerging biological preparations, have unique advantages of natural safety, immunomodulatory effects, flora-restoring abilities, and pathogenic bacteria-antagonizing effects and have the potential to help eradicate *H. pylori* biofilm cells [27]. Probiotics have been shown to inhibit pathogen biofilm formation by inhibiting pathogen growth, hindering pathogen adhesion and colonization, and interfering with the pathogen quorum sensing system [8]. Other studies have found that probiotics are able not only to inhibit pathogen biofilm formation but also to eradicate mature biofilms of bacteria such as *Escherichia coli* and *Enterococcus faecalis* [28,29]. Furthermore, there are many reports about probiotics such as *L. plantarum*, *L. rhamnosus* and *L. acidophilus* can antagonize *H. pylori* planktonic cells [30,31,32]. Additionally, these probiotics must tolerate the harsh environment of human gastric to provide an anti-*H. pylori* effect. Probiotics with high aggregation and hydrophobicity abilities are deemed to potentially colonize the human gastrointestinal environment [11]. Meanwhile, the pH in human stomach ranges from 1 to 4 and consists of pepsin, the candidate probiotics for *H. pylori* eradication must survive the extreme harsh environment [33]. In our study, we found that *L. plantarum* LN66 can survive at pH 2.0 simulated gastric juice for 2 h, and holds a moderate hydrophobicity and auto-aggregation capabilities (Supplementary Figures S2 and S3). Thus *L. plantarum* LN66 was selected for subsequent study. As shown in Figure 1, we found that CFS of LN66 was able to significantly eradicate the mature biofilm of *H. pylori* ATCC43504. Through a basic analysis of CFS effectiveness, we found that proteinase K enzymatic digestion had a significantly greater weakening effect on the eradication ability of CFS than neutralization, while heating has no significant effect on CFS eradication ability. Probiotics have been previously shown to be able to secrete lactic acid, short-chain fatty acids, bacteriocins, and other antagonistic antimicrobial substances to antagonize *H. pylori* [34]. Based on our results that the protein, polysaccharide, and pH value were significantly lower than MRS medium and reference strain (Supplementary Table S2), we tentatively speculate that the eradication effect of LN66 CFS on *H. pylori* biofilms may be mostly attributable to heat-stable short peptides, with some heat-stable organic acids playing a secondary role. In addition, as shown in Figure 2, the biofilm eradication ability of LN66 CFS was not very sensitive to low dilution factors; it still had strong eradication ability after being diluted 2- to 8-fold. This may be because CFS does not contain only one antibacterial substance. The additive effects of multiple antibacterial substances enable CFS to eradicate *H. pylori* biofilms at high dilutions. We consider *L. plantarum* LN66 as a potential applicable strain for improved eradication of *H. pylori* biofilms.

However, clinical results show that probiotics alone can merely reduce small loads of *H. pylori* in infected individuals and that the use of probiotics as adjuncts to supplement some antibiotic therapies may result in the desired effects [3]. In vitro experiments performed by Yang implied that *Bifidobacterium breve* YH68 CFS can improve the effectiveness of some antibiotics in inhibiting *Clostridium difficile* [16]. Soleymanzadeh et al. found that there are synergistic inhibitory effects between probiotics and tetracycline on clinical *Pseudomonas aeruginosa* strains [9]. Based on the finding that antibiotic sensitivity significantly decreased after *H. pylori* biofilm formation, and it is difficult for probiotic preparations alone to meet clinical needs, and there may be synergistic effects between probiotics and specific antibiotics. We investigated the differences in the effects of *L. plantarum* LN66 CFS, CLR and LVX acting separately or in combination on *H. pylori* mature biofilm eradication at the in vitro level. On the one hand, the FICs shows that the effect between CLR + CFS is antagonist, and the effect between LVX + CFS is additive. Additionally, the subsequent results showed that *L. plantarum* LN66 CFS significantly attenuated the eradication effect of CLR on *H. pylori* planktonic and biofilm cells, while there was some addictive effect between LN66 CFS and LVX. As shown in Figure 4 and Figure 10, for either planktonic cells or biofilm cells, the eradication effect of CLR was significantly reduced when CLR was used in combination with CFS. The opposite results were obtained for the LVX-CFS combined treatment group; although the eradication effect of LVX in combination with CFS was not significantly better than that of CFS alone, it was significantly better than that of LVX alone. Thus, the results obtained in the LVX-CFS combined treatment group imply that the interaction between LVX and CFS is not antagonistic. Therefore, the possibility of co-use exists. Moreover, from the results of whole-biofilm cell staining shown in Figure 7, we can infer that the effects of CFS and CLR on biofilm eradication were antagonistic, whereas the effects of CFS and LVX were additive. The SEM results shown in Figure 6 reflect that most of the cells in the surface layer of the *H. pylori* biofilm transformed from coccoid cells to spiral cells when CLR and CFS were combined, whereas most surface cells transformed from spiral cells to coccoid cells when LVX and CFS were combined. Previous studies have shown that taking the coccoid form is a way for *H. pylori* cells to resist external pressure; this form appears when there are antibiotics or nutrient deficiencies in the environment and can be accompanied by DNA structure changes and specific gene expression [35]. For example, when *H. pylori* ATCC43504 is treated with a 4 × MIC of lacticin A164, the majority of *H. pylori* cells transform from a spiral shape to a coccoid shape [36]. The transformation of surface biofilm cells from coccoid to spiral shapes may imply a decrease in the external pressure, which would also suggest that the eradication effects of LN66 CFS and CLR are antagonistic, whereas the eradication effects of LN66 CFS and LVX are additive. Furthermore, the CLSM results showed that the overall biofilm thickness and live cell thickness of the CLR + 1/2CFS group were higher than those of the rest of the groups, while the overall biofilm thickness and live cell thickness of the LVX + 1/2CFS group were lower than those of the rest of the groups. However, the overall thickness of the biofilm in the CLR and LVX groups was not significantly different before versus after combination with CFS, so the thickness of the biofilm may be influenced mainly by antibiotics. Thicker biofilms likely provide better defense for bacteria, while thinner biofilms may be helpful during clinical eradication. In addition, disruption of biofilms increases the risk of live bacteria dispersing into the surrounding environment and colonizing new surfaces, resulting in reinfection [37]. We determined the extent of live biofilm cell dispersion, as shown in Figure 9, and although there was an antagonistic effect between CLR and CFS in eradicating *H. pylori* biofilm, the combination was able to inhibit live biofilm cell dispersion as effectively as all the other treatments. We hypothesize that the improvement in efficacy exhibited by some probiotic supplementation therapies compared to traditional therapy in clinical trials and the lack of improvement exhibited by others is related to the different interactions between the probiotics and antibiotics used in the treatment.

The different interactions between CFS and CLR or LVX may be related to the different types of antibiotics and their mechanisms of action. CLR is a macrolide antibiotic that inhibits bacterial protein synthesis by reversibly binding to the 50S ribosomal subunit, and LVX is a fluoroquinolone antibiotic that targets chromosome replication [25,38]. Their differences may allow the two antibiotics to achieve different effects on *H. pylori* biofilm EPS when combined with CFS. Li found that treating *H. pylori* mature biofilms with CLR alone could reduce the protein content of EPS by 34.8% [5]. This phenomenon is consistent with our finding that the use of CLR alone was able to significantly reduce the protein content of *H. pylori* ATCC43504 mature biofilm EPS. However, when CLR was used in combination with CFS, its ability to reduce the EPS protein content was lost, and its ability to promote the EPS polysaccharide content was amplified. A thicker substrate, on the other hand, may have provided a better envelope in the CLR + 1/2CFS group, preventing killing by antimicrobial substances. LVX and CFS are more effective at disrupting biofilm EPS when combined than when used individually. Figure 5 shows that the combination of LVX and CFS reduced both the protein and polysaccharide content in EPS, and the change in matrix composition may have laid the foundation for the improved eradication effects of the antimicrobial substances. Therefore, CLR and CFS may not only be counteractive in eradicating *H. pylori* biofilms but also enhance the solidity of *H. pylori* biofilms when combined. On the other hand, although LVX and CFS in combination did not show an evident strong synergistic effect regarding biofilm eradication, they had a certain synergistic disrupting effect on the biofilm matrix.

The expression levels of *cagA* and *vacA*, two main virulence genes of *H. pylori*, are often associated with gastrointestinal dysfunction and are related to inflammation, cytotoxicity and apoptosis [39]. Urrutia-Baca et al. found that treating *H. pylori* with reuterin, secreted by *Lactobacillus reuteri*, can downregulate the expression of the *vacA* virulence gene [40]. Our results showed that CFS, CLR, and LVX, which had eradication effects on *H. pylori*, could significantly downregulate *H. pylori cagA* and *vacA* gene expression. There was a synergistic effect between CFS and these two antibiotics in inhibiting *cagA* and *vacA* expression. Thus, combination of LN66 with these two antibiotics may be helpful for alleviating the gastrointestinal dysfunction caused by *H. pylori* infection.

## 5. Conclusions

*H. pylori* biofilms are an obstacle to the clinical treatment of patients. Previous studies have shown that combinations of CLR and alginate lyase, LVX and bovine lactoferrin, and LVX and *Pistacia vera* L. oleoresin can inhibit *H. pylori* synergistically [41,42,43]. In our study, we investigated the different effects of CLR or LVX when used alone or in combination with *L. plantarum* LN66 CFS, which has anti-*H. pylori* capability, with regard to eradicating *H. pylori* mature biofilms. The results showed that combined treatment with CFS attenuated the eradication effect of CLR on *H. pylori* mature biofilms, while combined treatment with CFS promoted the disruptive effect of LVX on the *H. pylori* biofilm matrix. In addition, combined treatment with CFS and antibiotics had significant downregulatory effects on the expression levels of *H. pylori* biofilm virulence genes. Taking the clinical results of probiotic supplementation therapy into account, we believe that the use of *L. plantarum* LN66 as a supplementation may have the opposite effect during *H. pylori* treatment with CLR. The addition of *L. plantarum* LN66 may improve the eradication effects on *H. pylori* biofilms when using therapy involving LVX. However, the underlying mechanism by which LN66 CFS exhibits different interactions with CLR and LVX still needs to be thoroughly investigated, and further cell and animal experiments are needed to validate the mechanism. It is worth considering whether this probiotic–antibiotic mode of action is strain- and antibiotic-specific and determining which probiotics can act synergistically with antibiotics to support *H. pylori* clinical eradication.

## Figures and Tables

**Table 1:** Primers for qPCR.

Genes	Sequences (5′-3′)
*cagA*-F	GCAAGTGGTTTGGGTGGTGTAGG
*cagA*-R	CCGCCGAGATCATCAATCGTAGC
*vacA*-F	AGCGAGCGGGCGTTCTTTATTG
*vacA*-R	GGTATCCGTGCCAGCCTTAAACTC
16s rRNA-F	GGCGACCTGCTGGAACATTACTG
16s rRNA-R	CATCGTTTAGGGCGTGGACTACC

**Table 2:** Minimal inhibitory concentrations (MICs) of CLR and LVX.

Antibiotics	MICs (μg/mL)
CLR	0.063
LVX	0.250

**Table 3:** Fractional inhibitory concentrations (FICs) of antibiotics and CFS.

Combinations	FICs	Effect
CLR + CFS	3 (0.125 μg/mL, 12.5%)	Antagonist
LVX + CFS	1 (0.125 μg/mL, 6.25%)	Additive

**Table 4:** Thickness of biofilm observed by CLSM. The experimental data are expressed as the mean ± standard deviation (*n* = 3), and differences in thickness among live cells are expressed with different lowercase superscript letters (*p* < 0.05). Differences in thickness among dead cells are expressed with different capitalized superscript letters (*p* < 0.05).

Group	Live Cells (nm)	Dead Cells (nm)
Control	12.7 ± 1.5a	2.3 ± 0.6B
1/2CFS	6.7 ± 0.6c	1.7 ± 0.6B
CLR	7.3 ± 0.6c	5.7 ± 2.1A
CLR + 1/2CFS	11.7 ± 1.2ab	2.7 ± 0.6AB
LVX	9.3 ± 1.5bc	2.0 ± 1.0B
LVX + 1/2CFS	6.7 ± 1.2c	4.3 ± 1.5AB

## Data Availability

Data is contained within the article and supplementary material.

## Supplementary Materials

The following are available online at https://www.mdpi.com/2076-2607/9/2/424/s1, Figure S1: Phylogenetic relationship of LN66, Figure S2: Survival of LAB strains after 2 h in pH 2.0 simulated gastric acid, Figure S3: Auto-aggregation and hydrophobicity ability of four LAB strains, Table S1: Antagonistic abilities of LN66 CFS against five pathogens, Table S2: Protein, polysaccharide content and pH of LN66 CFS.
