# Supplementary material for: In Vitro Effects of Lactobacillus plantarum LN66 and Antibiotics Used Alone or in Combination on Helicobacter pylori Mature Biofilm
Source: Microorganisms. 2021 Feb 18;9(2):424. doi: 10.3390/microorganisms9020424 (PMC7923053; doi:10.3390/microorganisms9020424)
Supplement: Supplementary file 1 [file microorganisms-09-00424-s001.zip › Supplementary materials/Total Supplementary materials.docx]

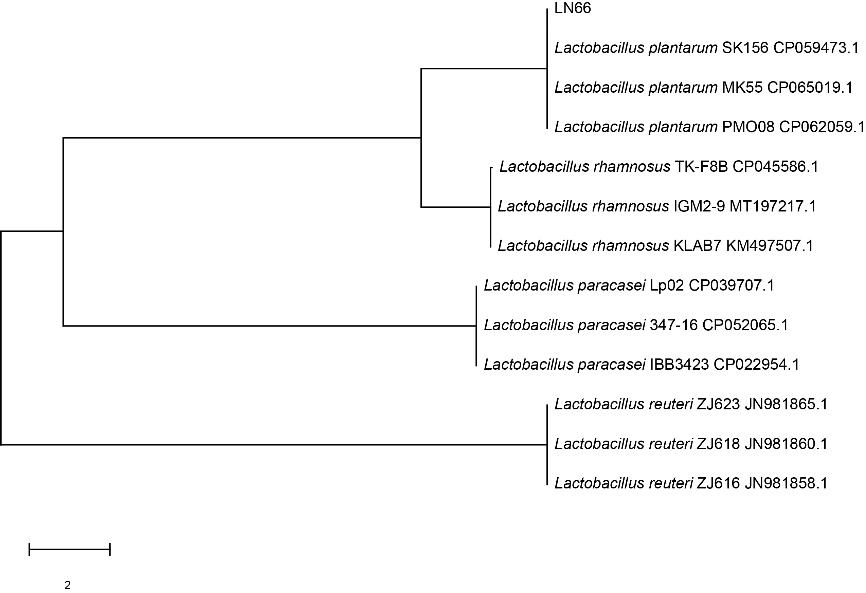


Supplementary Figure S1 Phylogenetic relationship of LN66 based on maximum likelihood analysis of the 16S rRNA gene, the evolutionary distances are calculated using the Tamura-Nei model. The scale bar represents 2-nucleotide substitutes per position.


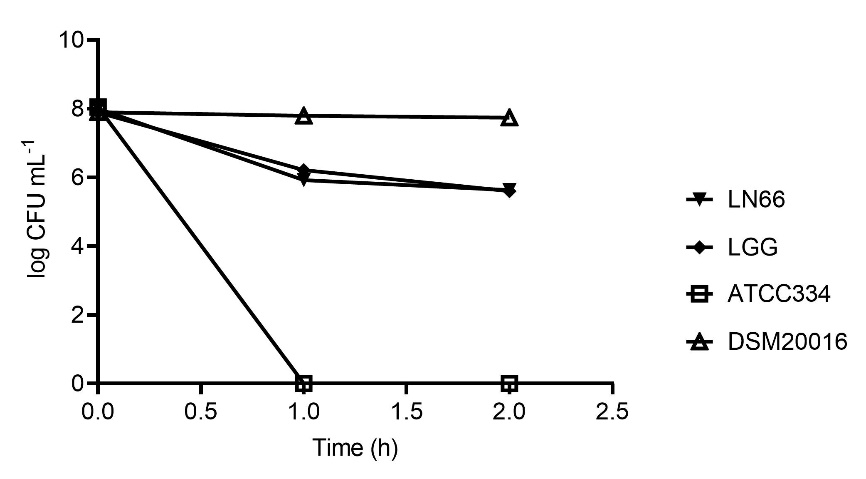


Supplementary Figure S2 Survival of LAB strains after 2 hours in pH 2.0 simulated gastric acid. Experimental data are expressed as mean ± standard deviation (n=3), and groups marked with different superscript letters indicate statistically significant differences (P ＜ 0.05).


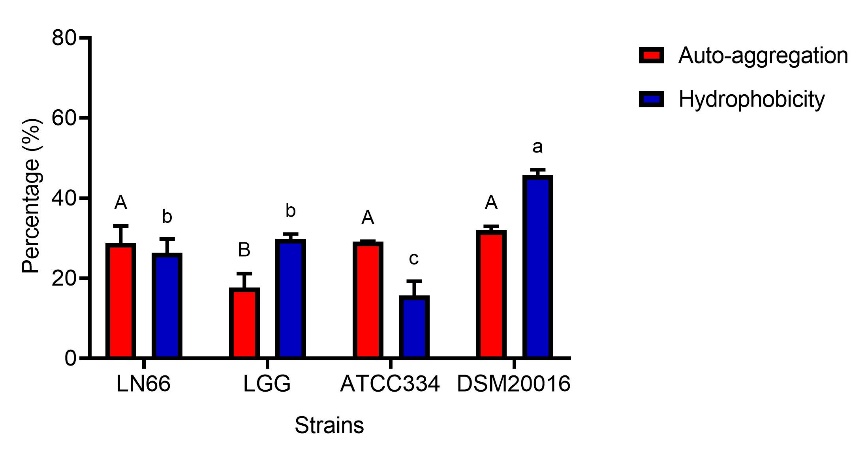


Supplementary Figure S3 Auto-aggregation and hydrophobicity ability of four LAB strains. Experimental data are expressed as mean ± standard deviation (n=3), and groups marked with different superscript letters indicate statistically significant differences (P ＜ 0.05).

Supplementary Table S1 Antagonistic abilities of LN66 CFS against five pathogens. Experimental data are expressed as mean ± standard deviation (n=3), and groups marked with different superscript letters indicate statistically significant differences (P ＜ 0.05).

|  | Average ZOI (mm) | | | | |
| --- | --- | --- | --- | --- | --- |
|  | *H. pylori* | *E. coli* | *S. aureus* | *S. sonnei* | *S. tyhimurium* |
| LN66 | 7.2±0.3^a^ | 7.5±0.1^a^ | 9.4±0.5^a^ | 8.2±0.2^a^ | 7.4±0.1^a^ |
| LGG | 4.6±0.2^b^ | 7.2±0.1^a^ | 8.6±0.3^ab^ | 7.6±0.1^b^ | 7.4±0.2^a^ |
| ATCC334 | 1.9±0.3^d^ | 7.3±0.1^a^ | 8.0±0.1^b^ | 7.7±0.1^b^ | 7.5±0.1^a^ |
| DSM20016 | 3.5±0.4^c^ | 7.3±0.2^a^ | 8.3±0.2^b^ | 7.8±0.3^b^ | 7.8±0.3^a^ |
| MRS | 0^e^ | 0^b^ | 0^c^ | 0^c^ | 0^b^ |

Supplementary Table S2 Protein, polysaccharide content and pH of LN66 CFS. Experimental data are expressed as mean ± standard deviation (n=3), and groups marked with different superscript letters indicate statistically significant differences (P ＜ 0.05).

| LAB strain | Protein content (μg/mL) | Polysaccharide (mg/mL) | pH |
| --- | --- | --- | --- |
| LN66 | 133.02±0.75^d^ | 17.01±4.11^b^ | 3.49±0.02^c^ |
| LGG | 138.31±0.87^bc^ | 22.38±6.47^b^ | 3.85±0.02^b^ |
| ATCC334 | 136.08±0.35^c^ | 35.90±3.46^a^ | 3.83±0.01^b^ |
| DSM20016 | 139.48±0.17^b^ | 15.49±0.79^b^ | 3.82±0.02^b^ |
| MRS | 144.99±1.53^a^ | 35.74±0.98^a^ | 5.88±0.02^a^ |
