# Supplementary figures and images for: In Vitro Effects of Lactobacillus plantarum LN66 and Antibiotics Used Alone or in Combination on Helicobacter pylori Mature Biofilm
Source: Microorganisms. 2021 Feb 18;9(2):424. doi: 10.3390/microorganisms9020424 (PMC7923053; doi:10.3390/microorganisms9020424)

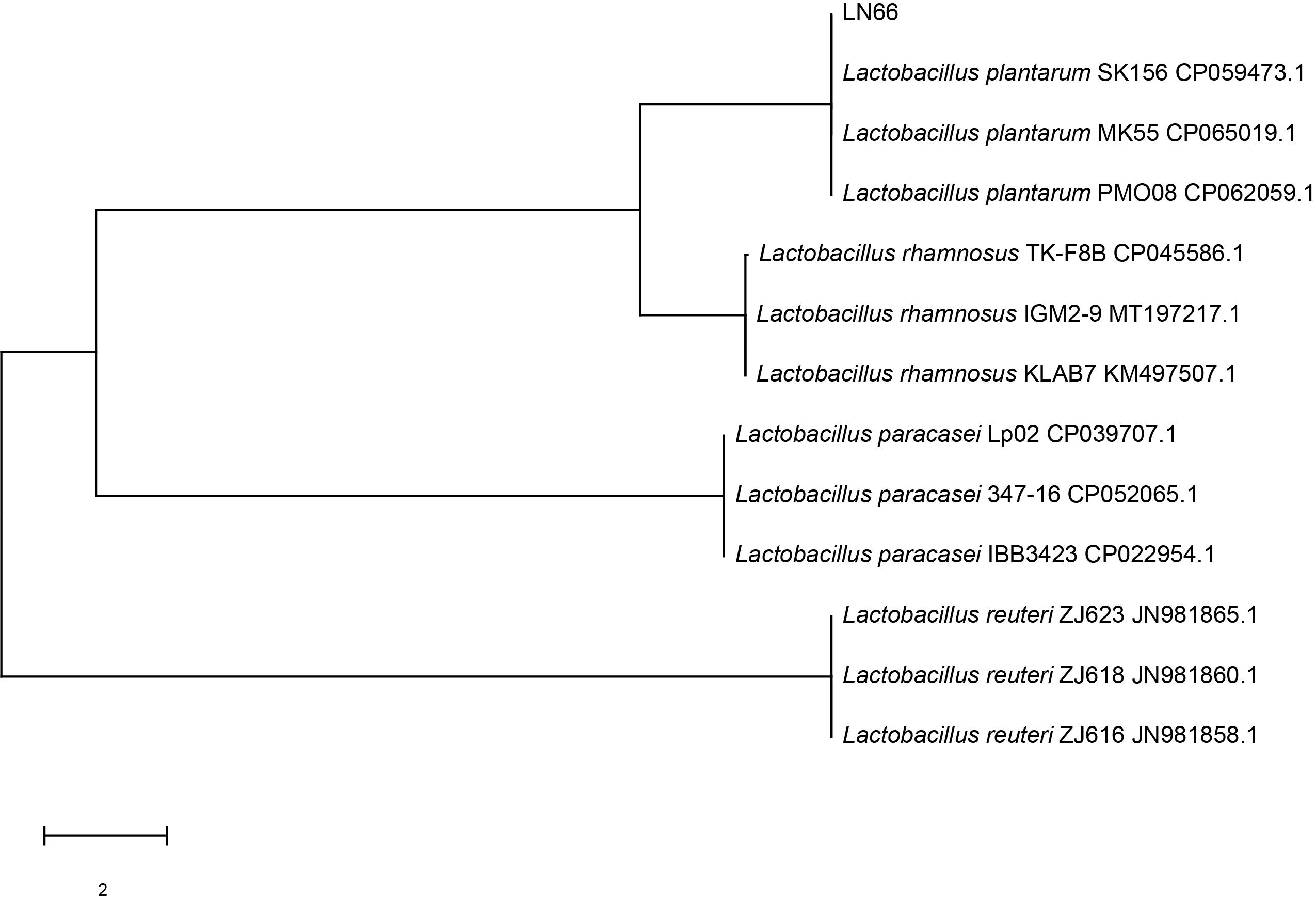

Supplement: Supplementary file 1 [file microorganisms-09-00424-s001.zip › Supplementary materials/Supplementary Figure S1/Supplementary Figure S1.jpg]

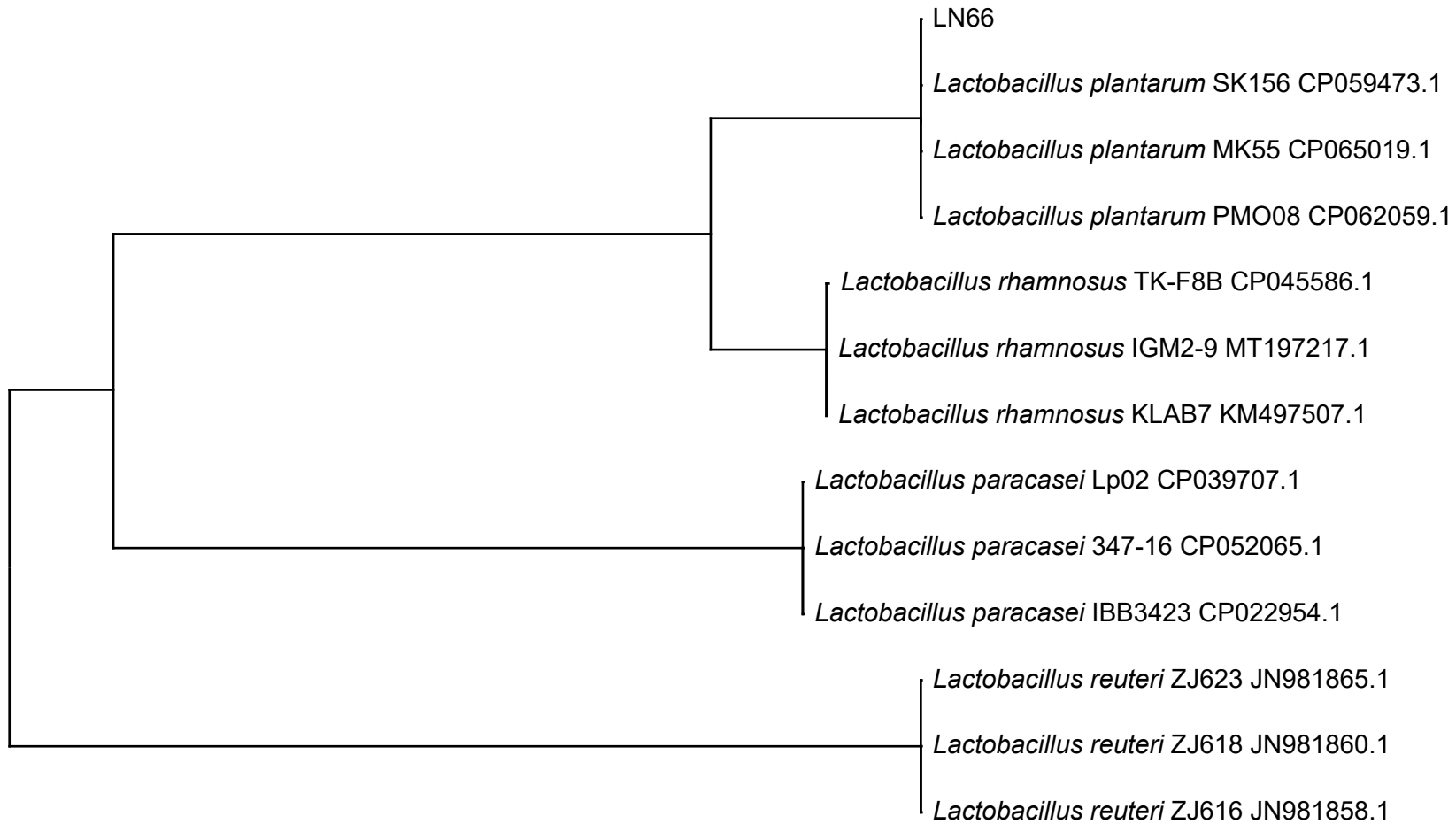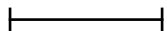

Supplement: Supplementary file 1 [file microorganisms-09-00424-s001.zip › Supplementary materials/Supplementary Figure S1/Supplementary Figure S1.pdf]

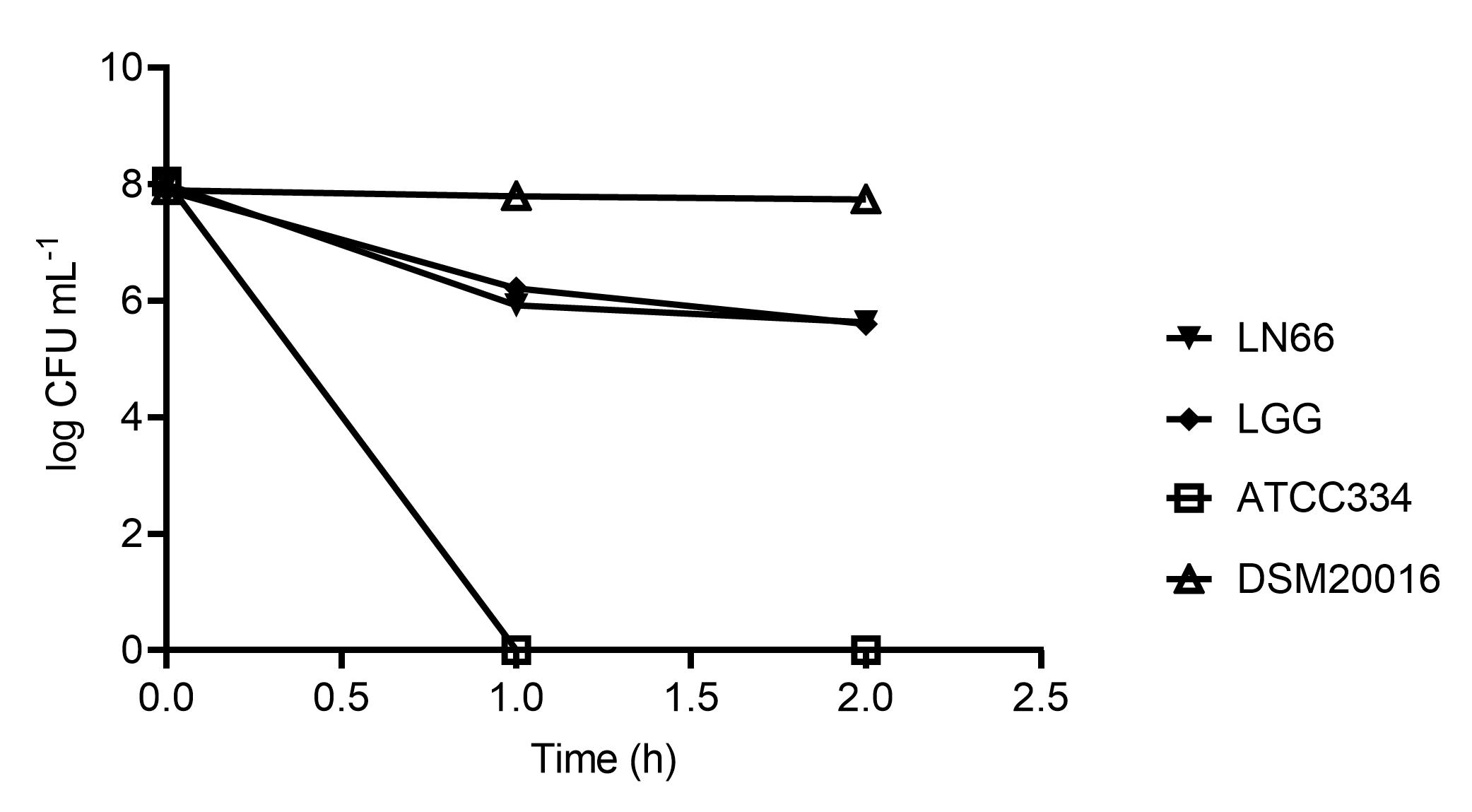

Supplement: Supplementary file 1 [file microorganisms-09-00424-s001.zip › Supplementary materials/Supplementary Figure S2/Supplementary Figure S2.jpg]

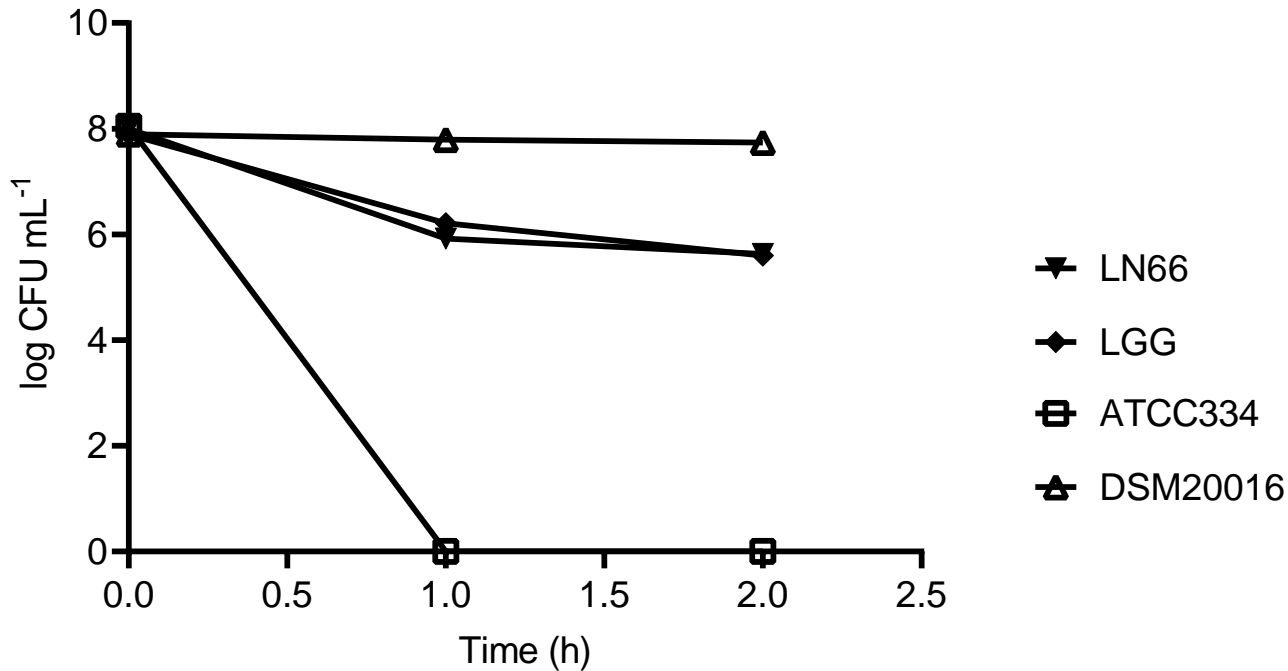

Supplement: Supplementary file 1 [file microorganisms-09-00424-s001.zip › Supplementary materials/Supplementary Figure S2/Supplementary Figure S2.pdf]

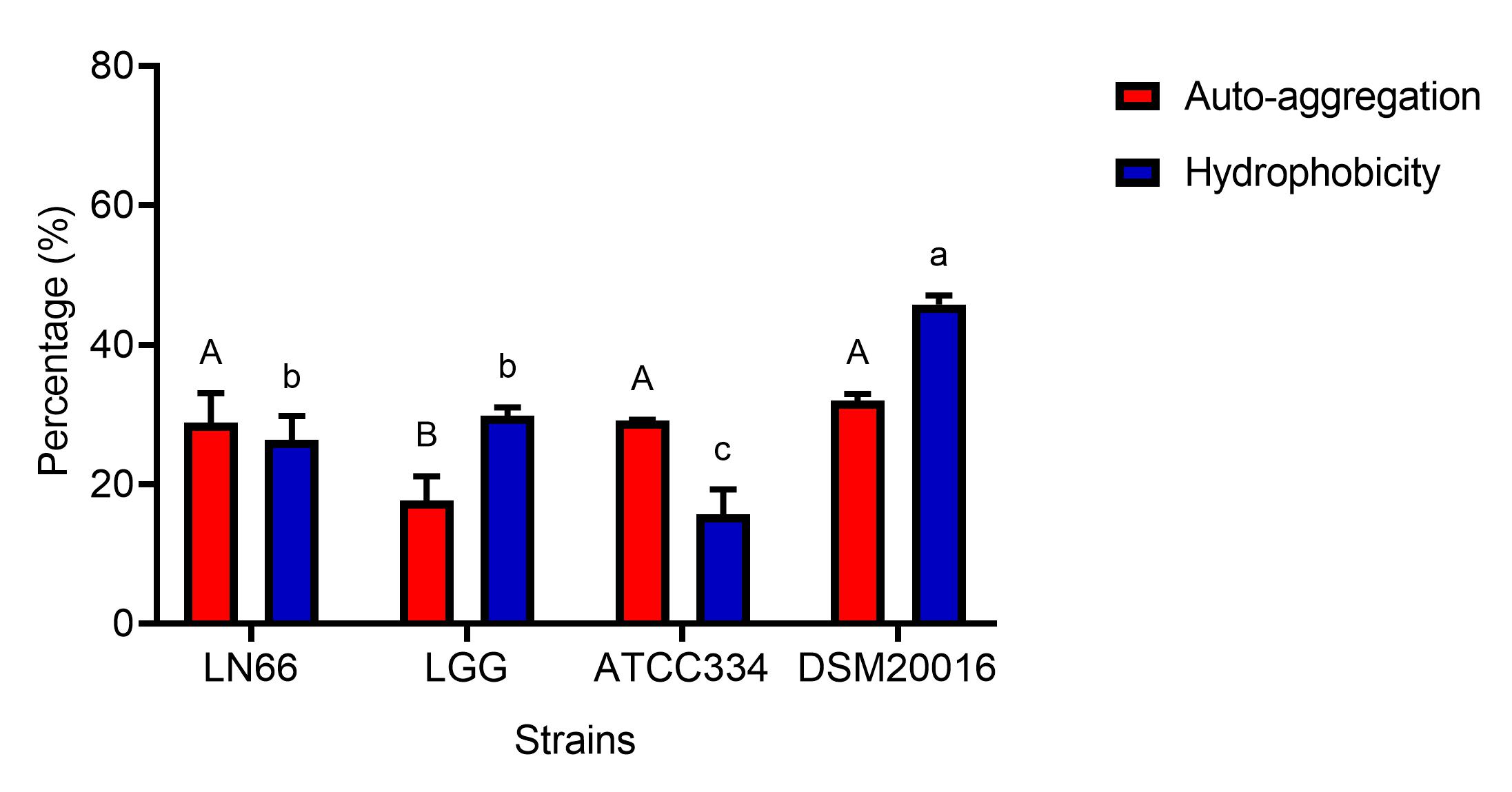

Supplement: Supplementary file 1 [file microorganisms-09-00424-s001.zip › Supplementary materials/Supplementary Figure S3/Supplementary Figure S3.jpg]

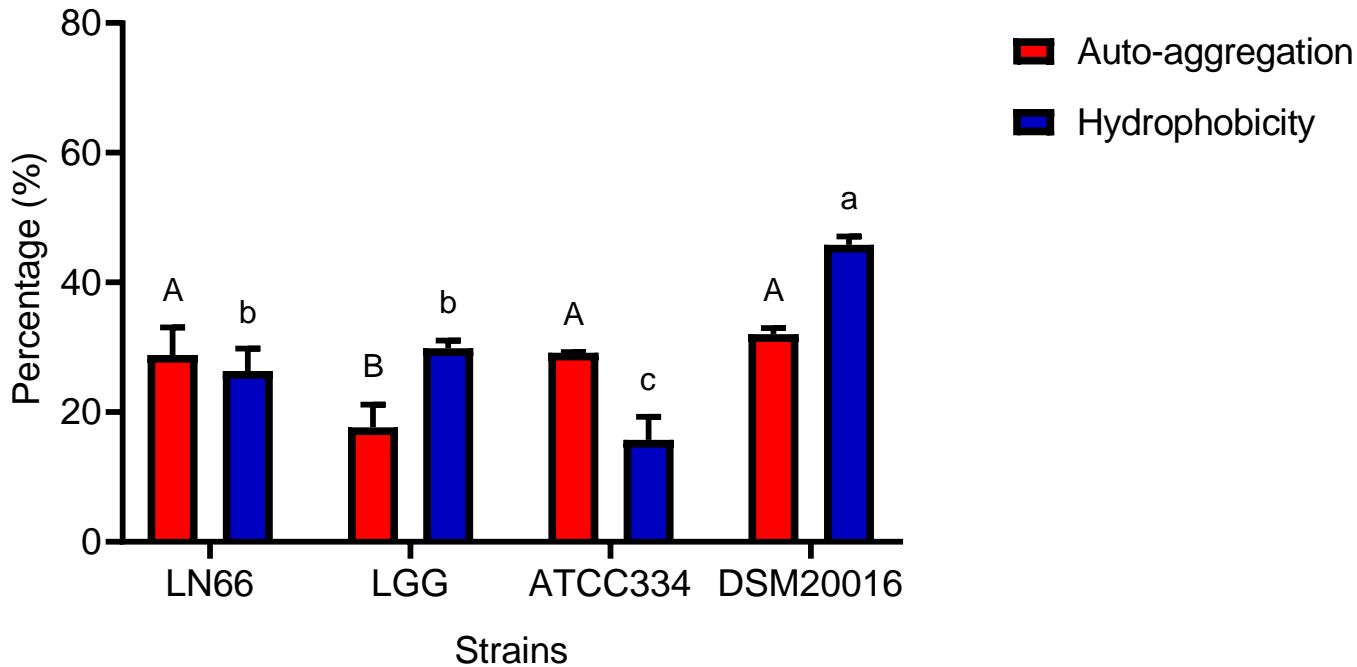

Supplement: Supplementary file 1 [file microorganisms-09-00424-s001.zip › Supplementary materials/Supplementary Figure S3/Supplementary Figure S3.pdf]
